# Dissecting the antibacterial functions of the T6SS-2 cluster in *Xanthomonas oryzae* for environment and plant protection

**DOI:** 10.1128/aem.01021-25

**Published:** 2025-09-19

**Authors:** Zhi-Min Tan, Xin Zheng, Jingtong Su, Jin-Sheng Liu, Xiaoye Liang, Tong-Tong Pei, Tao Dong

**Affiliations:** 1 State Key Laboratory of Microbial Metabolism, Joint International Research Laboratory of Metabolic and Developmental Sciences, School of Life Sciences and Biotechnology, Shanghai Jiao Tong University 12474, Shanghai, China; 2 Department of Immunology and Microbiology, School of Life Sciences, Southern University of Science and Technology 255310, Shenzhen, China; The University of Tennessee Knoxville, Knoxville, Tennessee, USA

**Keywords:** type VI secretion system, animal and plant pathogens, biocontrol-like agent

## Abstract

Plant pathogens pose a serious threat to global food security, and the excessive use of chemical pesticides has raised significant ecological concerns. Harnessing microbial competition presents a promising green technology for crop protection. In this study, we examined the functions of the type VI secretion system (T6SS), a crucial microbial competition tool, in *Xanthomonas oryzae* pv. *oryzae* strain PXO99A. PXO99A exhibited significant antibacterial activities dependent on its T6SS-2 cluster. Using genome, secretome, and functional analysis, we systematically predicted T6SS-associated effectors, detected the expression of five putative effectors, and functionally validated the toxicity of a VasX_N family effector PXO_00500 and its immunity protein PXO_RS08595. We further show that PXO99A uses its T6SS-2 to kill a broad range of plant and animal pathogens *in vitro* and *in planta*. Inactivation of the T3SS functions abolished virulence but had little effect on the T6SS-2-dependent antibacterial activities. Finally, we demonstrated that the avirulent T3SS-defective mutant is effective in protecting tomatoes from *Pseudomonas syringae* co-infection. Collectively, these results highlight an effective biocontrol strategy for plant protection.

**IMPORTANCE:**

The growing concerns over the toxicity, environmental impact, and resistance associated with chemical pesticides underscore the urgent need for alternative pathogen management strategies. In this study, we introduce an innovative approach of “turning waste into treasure” by repurposing plant pathogens as biocontrol-like agents. By elucidating the virulence and antimicrobial functions of *Xanthomonas oryzae*, we demonstrate that an avirulent mutant can employ its T6SS to effectively combat a broad spectrum of human and plant pathogens. Furthermore, its ability to protect tomato plants underscores its significant potential for sustainable agricultural practices.

## INTRODUCTION

Global food sustainability faces increasing challenges due to the continuous growth of the world population (1). Plant diseases, caused by insect pests and plant pathogens, significantly affect agricultural productivity, leading to annual crop losses of 10%–15% globally and economic losses amounting to hundreds of billions of dollars (2). The widespread reliance on chemical pesticides to control plant pathogens has led to serious environmental and health concerns, including the contamination of food, soil, water, and the broader ecosystem (3). Pesticide residues are frequently detected in food products, water sources, and soils, posing substantial risks to public health. Many chemical pesticides are known to be carcinogenic, cytotoxic, and mutagenic, further raising concerns about their safety (4). The growing resistance of pathogens to pesticides, combined with a decrease in the development of new chemical pesticides, underscores the pressing need for alternative, sustainable pathogen management strategies.

Biocontrol, using natural microorganisms to manage plant diseases, offers a viable alternative to chemical pesticides (5–12). For example, *Pseudomonas mosselii* strain 923 produces pseudoiodinine, which inhibits the growth of the rice pathogens *X. oryzae* and *Magnaporthe oryzae* (13). *Streptomyces hygroscopicus* secretes rapamycin, which inhibits the growth of fungal pathogen *Fusarium graminearum* (14). Moreover, *Pseudomonas putida* ISOF uses the type IVB secretion system to protect tomato plants from *Ralstonia solanacearum* (15), and *Acidovorax citrulli* exhibits strong bactericidal activity against various pathogens via the type VI secretion system (T6SS) (16). These findings highlight the potential for developing environmentally friendly antimicrobial agents by leveraging bacterial antagonism, presenting a promising approach to enhancing agricultural sustainability and reducing reliance on chemical pesticides.

Bacterial antagonism can be carried out by a number of protein machines that allow bacteria to occupy ecological niches and gain competitive advantages (17). Among these, the T6SS in gram-negative bacteria is a prominent apparatus that injects effector proteins directly into a broad range of neighboring prokaryotic and eukaryotic cells (18, 19). Structurally, the T6SS resembles an inverted T4 bacteriophage and is composed of a complex with at least 13 conserved components, including the membrane complex (TssJLM), baseplate (TssEFGK), and a double-tubular structure formed by an outer sheath (VipAB or TssBC) and an inner needle-like tube made of Hcp proteins (18–21). Assembly begins with the formation of the membrane complex, which anchors the T6SS to the cell envelope (19). The baseplate is then recruited to the membrane complex, initiating the assembly of a sharpening spike composed of a VgrG trimer and a cone-shaped PAAR protein, which is followed by the assembly of the inner Hcp tube and the outer contractile sheath (22–24). The T6SS is capable of ejecting effector proteins within milliseconds, with sufficient force to penetrate the cell envelopes of bacteria and fungi (25–28). This system delivers a diverse range of effector proteins that can target bacterial, fungal, plant, and animal cells (20, 29–31). The versatility of the T6SS makes it a promising tool for bacterial competition in diverse environments.

*X. oryzae* is a gram-negative bacterium responsible for bacterial blight in rice, a disease that causes significant economic losses, particularly in Southeast Asia and West Africa (32). *Xanthomonas* species employ a diverse array of molecular strategies to enhance pathogenicity and survival, including multiple secretion systems along with its associated effectors (32, 33). Although not all secretion systems are directly implicated in virulence, they collectively contribute to the overall fitness and adaptability of the pathogen in diverse environmental conditions. Notably, the T3SS secretes effector proteins that target plant hosts and modulate their immune responses (34, 35), playing critical roles in suppressing host defenses and promoting disease progression. In contrast, the role of the T6SS in *Xanthomonas* species is less understood. In *X. oryzae* pv. *oryzicola* strain GX01, the T6SS-2 cluster, but not the T6SS-1, exhibits antibacterial activity (36). However, another study in *X. oryzae* pv. *oryzae* strain PXO99A reported that neither T6SS1 nor T6SS2 displayed antibacterial activity (37).

In this study, we characterized the T6SS functions of PXO99A. Our findings reveal that the T6SS-2 in PXO99A exhibits broad antibacterial activity against both human and plant pathogens. Inactivation of the main virulence factor T3SS abolished the virulence of PXO99A to plants but did not affect its T6SS-2 antibacterial functions. Importantly, the T3SS-defective PXO99A mutant was capable of killing pathogens *in planta* in a T6SS-2-dependent manner. These results highlight the potential of leveraging T6SS-mediated antagonism for plant protection.

## RESULTS

### Secretome analysis and prediction of effectors

**Fig 1:**
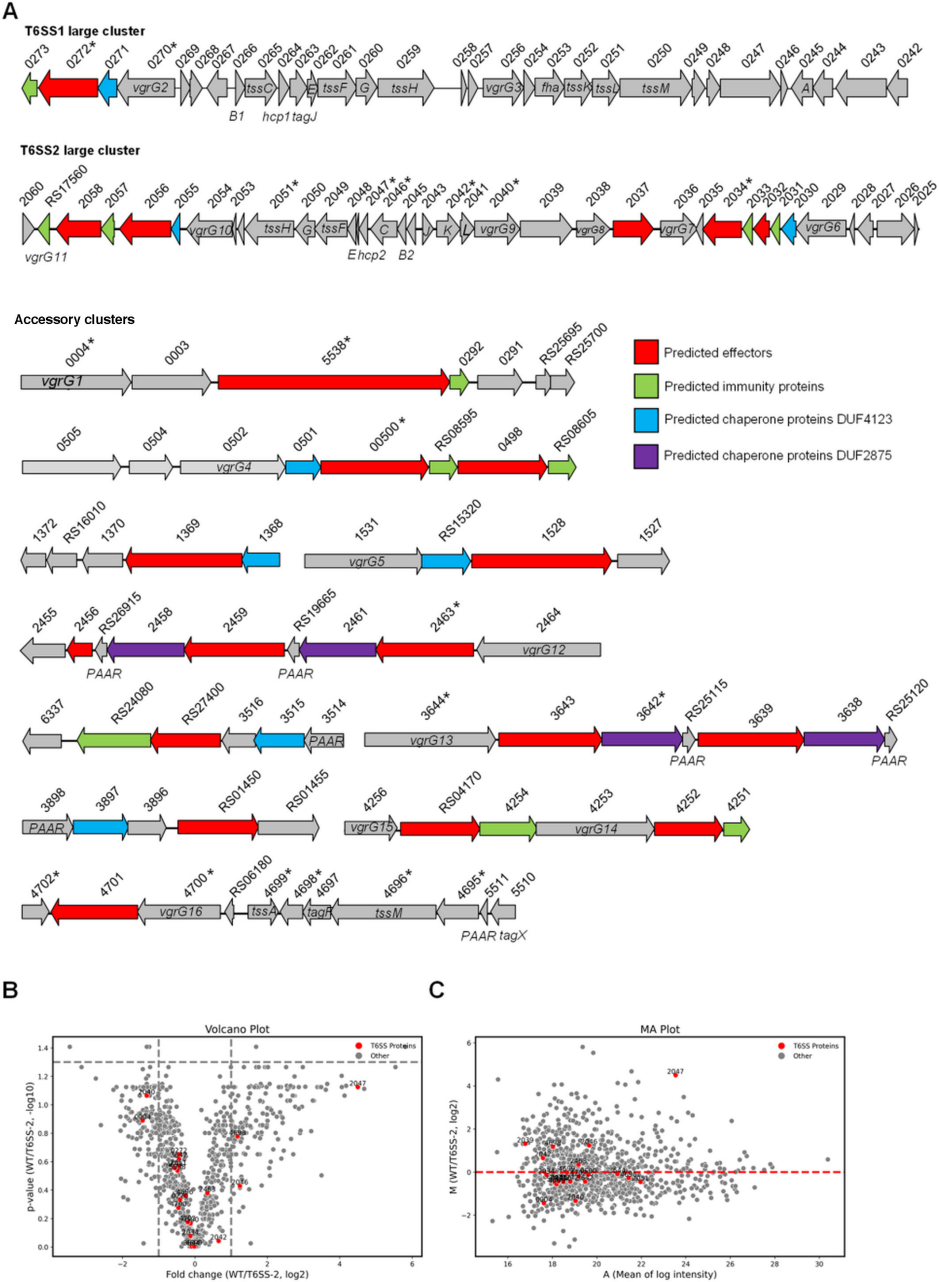
Identification of T6SS effector proteins. (**A**) Genomic organization of the *X. oryzae* PXO99A T6SS clusters, including T6SS-1, T6SS-2, and accessory clusters. (**B**) Volcano plot illustrating differentially secreted proteins in the comparison between WT (PXO99A) and Δ*tssB2* strains. (**C**) MA plot showing secreted proteins identified by mass spectrometry, comparing WT (PXO99A) and Δ*tssB2* strains. Secretome data are provided in Data Set S1 for **B** and **C. **In panels **A–C**, “0273” corresponds to PXO_00273, and “RS17560” refers to PXO_RS17560, with all other numbers representing their respective gene identifiers. The “*” symbol highlights proteins identified in both secretome analyses in panels **B** and **C**. In panels **B** and **C**, each dot represents a single protein, with T6SS proteins highlighted in red and other secreted proteins shown in gray.

In the PXO99A genome, two large clusters, T6SS-1 and T6SS-2, encode the majority of the proteins that make up the T6SS membrane complex and baseplate (Fig. 1A). Additionally, these clusters also contain genes for secreted structural components VgrG and Hcp. Specifically, the T6SS-1 cluster encodes VgrG2, VgrG3, and Hcp1, while the T6SS-2 cluster includes genes for six different VgrG paralogs and Hcp2. Based on the known genetic connections between VgrG, PAAR, chaperone proteins (DUF4123 and DUF2875 families), and effector proteins (24, 38, 39), we predicted a total of 10 accessory clusters that code for effectors. In total, 21 candidate effector genes were identified (Fig. 1A and Table S1).

To examine the secretion of these predicted effectors, we employed a secretome approach using LC-MS/MS analysis to compare the secreted proteins of the wild type and the T6SS-null Δ*tssB2* mutant grown to an OD_600_ of 1 under lab conditions. We detected 992 and 946 proteins in the wild type and the Δ*tssB2* mutant, respectively (Fig. 1B and C). Notably, Hcp2 (PXO_02047), the most abundant secreted protein of the T6SS, was enriched 26-fold in the wild-type sample (Fig. 1B and C). However, most other T6SS-associated proteins were not significantly changed under the conditions tested (Fig. 1B and C). This is likely attributable to a high level of cell autolysis because we also detected a substantial amount of cytosolic proteins, including ribosomal proteins and metabolic enzymes. Nonetheless, we did not detect any T6SS-1 structural proteins but found most T6SS-2 components, suggesting that the T6SS-2 but not the T6SS-1 is active. Five VgrG proteins and five predicted effectors were also detected (Fig. 1B and C).

### T6SS-2 exhibits active and bactericidal activities

**Fig 2:**
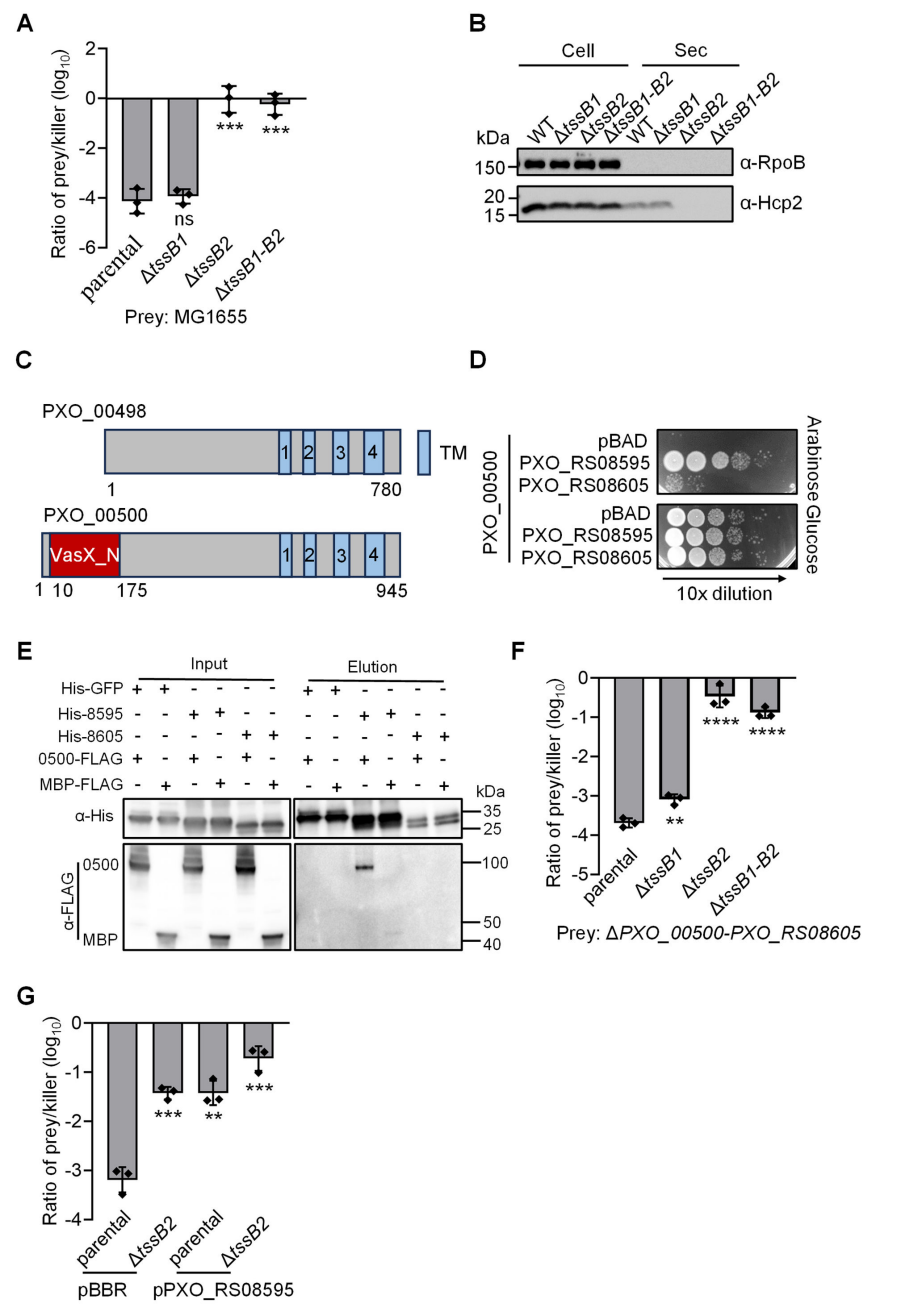
Functionality of the *X. oryzae* T6SS-2. (**A**) Competition assay of prey cells (*E. coli* MG1655) after co-incubation with killer strains *X. oryzae* (parental) and strains containing an inactivating deletion of the T6SS sheath components *tssB1* (T6SS-1), *tssB2* (T6SS-2), and Δ*tssB1-B2* double deletion (T6SS-1 and 2). (**B**) The secretion of Hcp2 in *X. oryzae*. The Hcp2 protein was detected by Western blot analysis using an anti-Hcp2 antibody. Detection of the RpoB was used as a control. The position of the molecular size marker (in kDa) is indicated. (**C**) Schematic diagram of the predicted PXO_00498 and PXO_00500 domain structure. For PXO_00498, TM, transmembrane, TM1 coordinates 560–577, TM2 coordinates 598–614, TM3 coordinates 634–657, and TM4 coordinates 704–726. For PXO_00500, VasX_N, VasX (pore-forming toxin) toxin N-terminal region; TM1 coordinates 724–741, TM2 coordinates 762–780, TM3 coordinates 800–821, and TM4 coordinates 868–890. Secondary structure is predicted by HAMMER. (**D**) Toxicity assay of PXO_00500 in *E. coli*. Serial dilutions of *E. coli* expressing PXO_00500 with either an empty vector (pBAD) or vectors encoding the immunity genes PXO_RS08595 or PXO_RS08605 were analyzed. To direct PXO_00500 to the periplasmic space, a Tat signal peptide was fused to its N-terminus. (**E**) Interaction of 0500 (PXO_00500) with 8595 (PXO_RS08595) or 8605 (PXO_RS08605). Pull-down analysis was performed using His-GFP (control), His-8595 or 8605, MBP-FLAG (control), and 0500-FLAG. (**F**) Competition assay of prey cells (Δ*PXO_00500-PXO_RS08605*) after co-incubation with killer strains PXO99A (parental) and strains containing an inactivating deletion of the T6SS structure components *tssB1* (T6SS-1), *tssB2* (T6SS-2), and Δ*tssB1-B2* double deletion (T6SS-1 and 2). (**G**) Competition assay of WT and the T6SS-null Δ*tssB2* mutant against the effector–immunity deletion mutant Δ*PXO_00500-PXO_RS08605* complemented with an empty vector (pBBR) or a vector expressing the immunity protein PXO_RS08595 as indicated. The data point indicates the relative survival of prey cells attacked by WT compared with that by the T6SS-2 mutant. Error bars indicate standard deviation across three biological replicates. Significance was determined by a two-tailed Student’s *t*-test (*P < 0.05, **P < 0.01, ***P < 0.001, and ****P < 0.0001; ns, not significant).

To evaluate the T6SS functions in *X. oryzae* PXO99A, we performed interspecies competition assays using *Escherichia coli* strain MG1655 as the prey. Wild-type PXO99A exhibited strong bactericidal activity, significantly outcompeting *E. coli*. In contrast, the Δ*tssB2* mutant demonstrated significantly impaired killing activity, whereas the *tssB1* mutant retained wild-type killing levels (Fig. 2A). These findings indicate that the T6SS-2 plays a critical role in the antibacterial activity for interspecies competition.

We also evaluated the expression and secretion of Hcp2, the inner tube component whose secretion level is a hallmark of T6SS activity. Using a custom polyclonal antibody, we found that Hcp2 was well expressed and secreted in the T6SS-2-dependent manner (Fig. 2B). These results are consistent with our proteomic results, confirming that the T6SS-2 is active under the lab conditions tested.

### PXO_00500 and PXO_RS08595 form an effector-immunity pair

Of these effector-encoding clusters, we noticed a cluster encoding a duplicated effector pair, PXO_00500 and PXO_00498, sharing 92% identity in amino acid sequence (Fig. S1). The PXO_00500 protein contains a conserved VasX_N domain (IPR046864), which is originally found in the T6SS antibacterial/anti-eukaryotic effector VasX from *Vibrio cholerae* (Fig. 2C) (40, 41). Their neighboring genes, PXO_RS08595 and PXO_RS08605, encode putative immunity proteins of the T6SS_Burk_ExIF superfamily and also share high sequence similarity (100% coverage and 68% identity) (Fig. S2).

Given the high sequence similarity between PXO_00500 and PXO_00498, we focused on the toxicity of PXO_00500. The expression of PXO_00500 in the periplasm of *E. coli*, using a twin-arginine translocation (Tat) signal sequence (42), induced significant toxicity, which was markedly reduced by co-expression with PXO_RS08595 or PXO_RS08605, with PXO_RS08595 almost completely neutralizing the toxicity (Fig. 2D). Pull-down analysis demonstrated direct interaction between PXO_00500 and PXO_RS08595 (Fig. 2E). In contrast, periplasmic expression of PXO_00498 exhibited no cytotoxicity in *E. coli*, despite detectable expression by Western blot analysis (Fig. S3A and B). Previous studies have shown that the *V. cholerae* cell wall-targeting effector TseH is non-toxic to *E. coli* but highly toxic to *Aeromonas dhakensis* due to T6SS immunity-protein-independent protective pathways in *E. coli* (42–44). To determine whether PXO_00498 exhibits similar host-specific toxicity, we expressed it with an N-terminal Sec signal peptide in *A. dhakensis*. This induced significant cytotoxicity, which was strongly suppressed by co-expression of the immunity protein PXO_RS08605 (Fig. S3C and D). These findings suggest that PXO_00498 and PXO_00500 are toxic effectors and that PXO_00498/PXO_RS08605 and PXO_00500/PXO_RS08595 function as effector-immunity pairs.

Given the observed cross-protection between PXO_RS08595 and PXO_RS08605, we constructed a quadruple knockout mutant lacking PXO_00500, PXO_RS08595, PXO_00498, and PXO_RS08605, designated as Δ*PXO_00500-PXO_RS08605*. This mutant exhibited a significantly reduced survival rate in competition assays against the wild type and Δ*tssB1* mutants compared to the Δ*tssB2* and Δ*tssB1-B2* mutants (Fig. 2F). Supplementing the Δ*PXO_00500-PXO_RS08605* mutant with PXO_RS08595 significantly restored prey cell survival (Fig. 2G). These findings suggest that PXO_00500 functions as a T6SS effector protein whose secretion is dependent on the T6SS-2 cluster.

**Fig 3:**
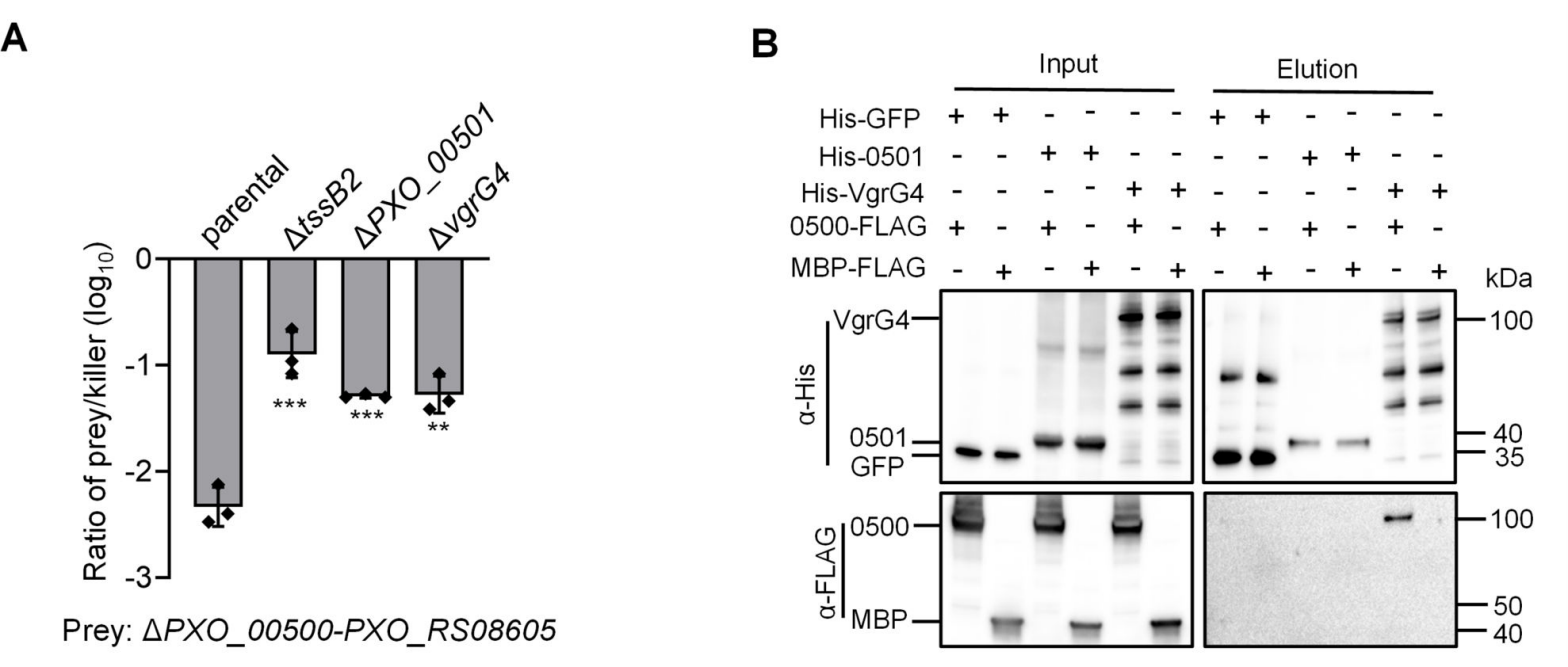
Secretion of PXO_00500 requires chaperone PXO_00501 and VgrG4. (**A**) Competition analysis of Δ*PXO_00501* (chaperone) and Δ*vgrG4*. Killer strains are indicated, and the prey strain is the Δ*PXO_00500-PXO_RS08605* mutant. (**B**) Interaction of PXO_00500 with PXO_00501 or VgrG4. Pull-down analysis was performed using His-GFP (control), His-0501 or His-VgrG4, MBP-FLAG (control), and 0500-FLAG. Error bars indicate the mean ± s.d. of three biological replicates, and significance was calculated using a two-tailed Student’s *t*-test (**P < 0.01 and ***P < 0.001).

The genes upstream of PXO_00498 and PXO_00500 encode the structural protein VgrG4 (PXO_00502) and the chaperone protein PXO_00501 (Fig. 1A). To determine whether the secretion of PXO_00500 depends on VgrG4 and PXO_00501, we constructed deletion mutants of these two genes. Competition assays revealed a significant reduction in the killing ability of these two mutants compared to the wild-type strain (Fig. 3A). Furthermore, pull-down assays confirmed that VgrG4 interacts with PXO_00500 (Fig. 3B). Together, these results indicate that VgrG4 and PXO_00501 are required for PXO_00500 secretion.

### T6SS-2 mediates competition against plant and animal pathogens

**Fig 4:**
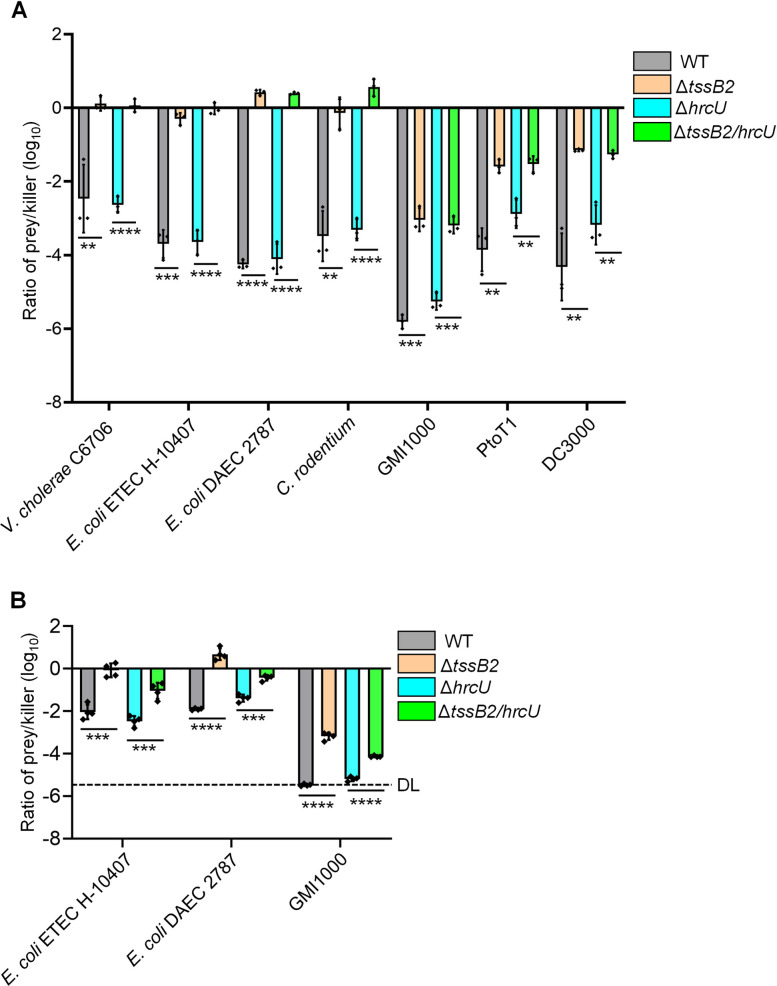
T6SS-dependent killing of animal and plant pathogens *in vitro* and *in planta*. (**A**) Competition assay with pathogens that could contaminate edible plants and important plant pathogens. *Ralstonia solanacearum* GMI1000, GMI1000; *Pseudomonas syringae* pv. *tomato* PtoT1, PtoT1; *P. syringae* pv. *tomato* DC3000, DC3000. WT, *X. oryzae* PXO99A wild type; Δ*tssB2*, T6SS-2-null strain; Δ*hrcU*, T3SS-null strain; Δ*tssB2/hrcU*, a mutant in which both T3SS and T6SS are inactive. (**B**) *In planta* competition assay with *X. oryzae* PXO99A against the animal pathogens *E. coli* ETEC H-10407, *E. coli* DAEC 2787, and the phytopathogen GMI1000. DL, detection limit. Error bars indicate the mean ± s.d. of three biological replicates, and significance was calculated using a two-tailed Student’s *t*-test (**P < 0.01, ***P < 0.001, and ****P < 0.0001).

Next, we investigated the antibacterial efficacy of T6SS-2 against a diverse array of human and plant pathogens, the former being significant for foodborne diseases due to vegetable contamination. Using competition assays, we found that T6SS-2 exerts significant bactericidal activity against human pathogens, *Vibrio cholerae* C6706, *enterotoxigenic Escherichia coli* (ETEC) H-10407, diffusely adherent *Escherichia coli* (DAEC) 2787, and a mouse-specific pathogen *Citrobacter rodentium*, as well as plant pathogens *R. solanacearum* GMI1000, *P. syringae* pv. *tomato* PtoT1, and *P. syringae* pv. *tomato* DC3000 (Fig. 4A).

Because the T3SS is crucial for the virulence of PXO99A (45), we also tested the effect of T3SS on the T6SS-2-mediated killing functions by deleting the *hrcU* gene, encoding the export apparatus (46). Using T3SS and T6SS-2 knockout mutants, we found that the killing ability of the T3SS-inactivated mutant was comparable to that of the wild type, indicating that T6SS-mediated antibacterial activity does not require the T3SS (Fig. 4A).

To determine whether T6SS enhances the competitive fitness of PXO99A in plants, we performed competition assays using a *Nicotiana benthamiana* infection model (27). We compared the wild-type PXO99A with T3SS-null (Δ*hrcU*) and T6SS-2-null mutants in their ability to kill the animal pathogens *E. coli* ETEC H-10407 and *E. coli* DAEC 2787, as well as the plant pathogen *R. solanacearum* GMI1000. *In planta* competition revealed that both the wild type and the T3SS-null mutant (Δ*hrcU*) significantly inhibited the growth of *E. coli* ETEC H-10407, *E. coli* DAEC 2787, and *R. solanacearum* GMI1000 in plants (Fig. 4B). These results suggest that the T6SS-2 of PXO99A is functional and enhances its fitness *in planta*.

### A T3SS-inactivated *X. oryzae* mutant protects tomato plants from *P. syringae* infection

**Fig 5:**
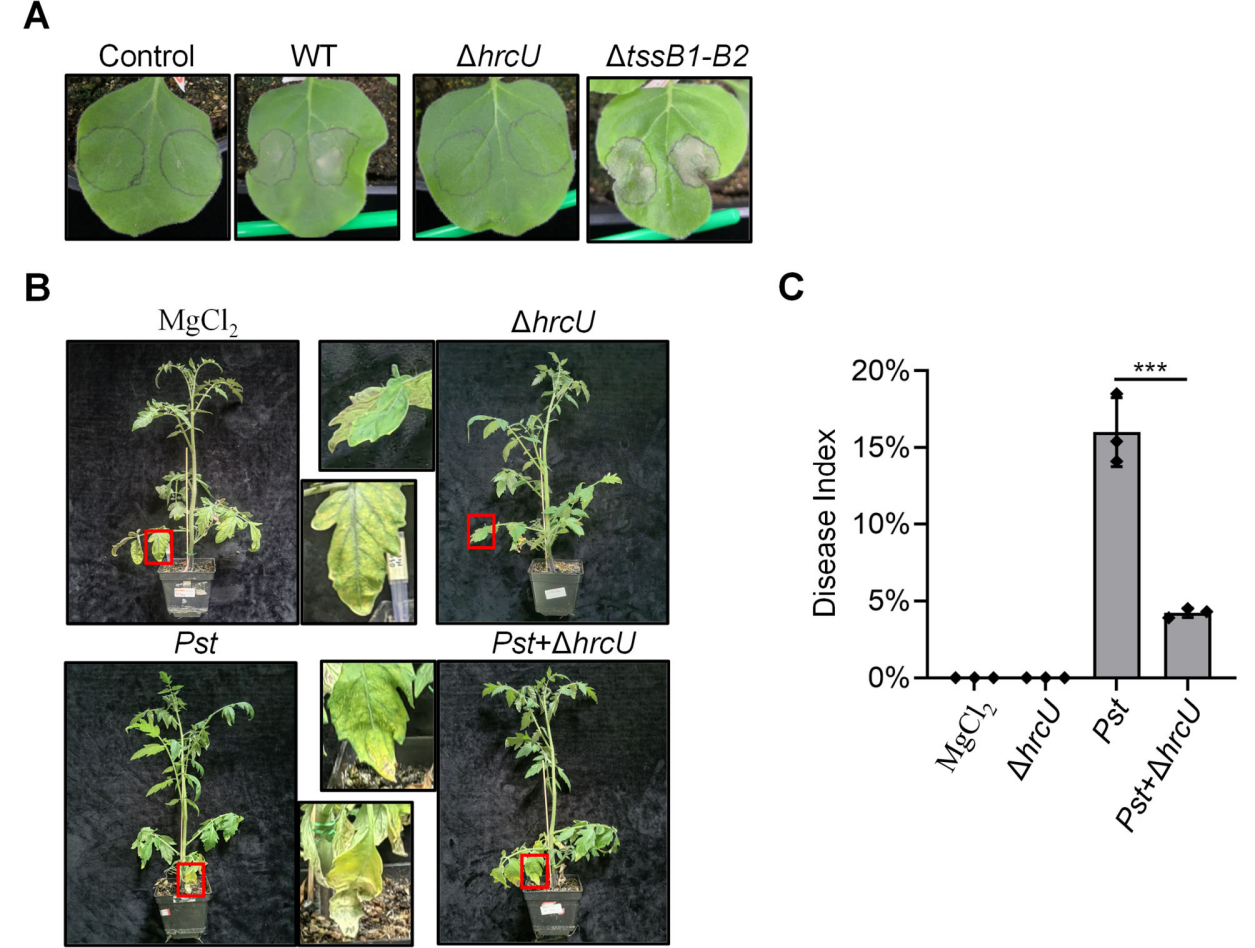
*X. oryzae* T3SS-null (Δ*hrcU*) protects tomato plants from *P. syringae* infection. (**A**) Hypersensitivity response of the *X. oryzae* in *N. benthamiana*. WT and its derivatives inoculated with 4-week-old *N. benthamiana* and observed after 48 hours. WT, *X. oryzae* PXO99A wild type; Δ*tssB1-B2*, T6SS-null strain; Δ*hrcU*, T3SS-null strain; and control, 10 mM MgCl_2_. (**B**) *X. oryzae* inhibits infection by *P. syringae* on tomato plants. Four-week-old tomato Ac plants were sprayed with a T3SS-inactivated mutant*, P. syringae* pv. *tomato* DC3000 (*Pst*), and a mixture of the T3SS-inactivated mutant and *Pst*. Images were captured 11 days post-inoculation, with H_2_O used as a control. (**C**) Disease index was calculated at 11 days post-infection. Error bars indicate the mean ± s.d. of three biological replicates, and significance was calculated using a two-tailed Student’s *t*-test (***P < 0.001).

To test the effects of the T6SS and T3SS mutants on plant infection, we tested the ability of PXO99A to induce a hypersensitive response (HR) in *N. benthamiana* leaves. We infiltrated the backs of *N. benthamiana* leaves with cell suspensions of the wild type, T6SS-inactivated mutant, and T3SS-inactivated mutant. To exclude the possibility that the cell resuspension buffer affected HR, we used 10 mM MgCl_2_ as a negative control. Visual observations revealed that the wild type and the T6SS deletion mutant induced a strong HR response. In contrast, the T3SS-inactivated mutant (deleting the *hrcU* gene, thus named PXO99A^av^) had no damage similar to the control (Fig. 5A).

Finally, we tested whether we could harness the T6SS-active and avirulent PXO99A^av^ (Δ*hrcU*) for plant protection. We used *P. syringae* pv. *tomato* DC3000 as the pathogen and tomato *Ailsa Craig* as the plant infection model. The results showed that tomatoes sprayed with MgCl_2_ or the Δ*hrcU* alone did not develop any disease (Fig. 5B and C). In contrast, tomatoes sprayed with DC3000 alone developed the disease. Tomato plants inoculated with a mixture of DC3000 and Δ*hrcU* exhibited significantly reduced disease symptoms, indicating effective protection against infection (Fig. 5B and C).

## DISCUSSION

Pathogenic microorganisms inhabit complex and dynamic environments, where they encounter various challenges within the same ecological niche, including competition with other microorganisms for nutrients and habitat, as well as evasion of the host immune responses. To adapt to these pressures, bacteria have evolved specialized mechanisms and molecular tools to regulate their interactions with both the host and other microorganisms in their surroundings (47–50). In this study, we report the critical role of the T6SS-2 in PXO99A for interspecies competition. The T6SS-2 efficiently kills a broad spectrum of animal and plant pathogens, highlighting its versatility and potency as a microbial weapon. Moreover, the active function of T6SS-2 *in planta* suggests its potential in enhancing the bacterial fitness of PXO99A during plant colonization. Remarkably, a PXO99A T3SS-null mutant was able to protect tomatoes from *P. syringae* pv. *tomato* DC3000, presenting a promising approach for biocontrol and green agricultural practices.

Plant pathogens have evolved robust survival strategies within their native ecological niches. Research on *X. oryzae* has traditionally focused on its pathogenicity in rice and the resistance mechanisms rice plants employ to counter infection (51–53). However, the environmental adaptability of *X. oryzae* is relatively less understood. In this study, we predicted 21 T6SS effector proteins in PXO99A, reflecting a broad repertoire that likely supports *X. oryzae* in competing against a diverse array of competitors. Exploring these T6SS effectors will deepen our understanding of the role T6SS plays in the physiology and ecological adaptability of *Xanthomonas*.

Because the T6SS-dependent bactericidal activity generally requires close cell-cell contact for effector delivery, physical barriers such as biofilms or extracellular matrices may impede this interaction in natural environments. For example, *R. solanacearum* GMI1000 produces abundant extracellular polysaccharides (54), which could obstruct direct contact with PXO99A and thereby reduce its inhibitory effects (Fig. 4A and B). Additionally, T6SS assembly and effector secretion are energetically costly processes, requiring ATP hydrolysis for sheath dynamics (19). This may limit the fitness of T6SS-armed strains in resource-limited environments, potentially reducing their efficacy as biocontrol agents. Finally, T6SS activity is tightly regulated in response to environmental cues, including quorum-sensing signals, nutrient availability, and stress signals (55–57). Such regulation could lead to inconsistent antibacterial effects under fluctuating field conditions. While these constraints pose challenges, they also highlight opportunities for bioengineering optimization. For example, we recently developed a cell wall-lysing effector that acquired gram-positive cell targeting capability in a contact-independent manner (58), suggesting that similar approaches could be applied to enhance T6SS effectiveness.

A key limitation of this study is the inability to evaluate the protective role of avirulent *X. oryzae* in its native rice ecosystem, where ecological performance and long-term durability ultimately determine its value as a biocontrol agent. The intrinsic resistance of wild-type Xoo to its own T6SS effectors creates a barrier to such *in situ* testing. To overcome this barrier, the T6SS would need to be engineered to deliver heterologous antibacterial effectors that circumvent self-resistance. A second limitation is that the protective effect of the T6SS depends on stable colonization of host tissues, a process likely shaped by host genotype, colonization dynamics, and the surrounding microbiome. Future efforts to engineer strains that can persist in diverse host environments will strengthen their utility as biocontrol agents.

The use of bacterial competition to manage plant pathogens has been demonstrated through numerous studies. Traditional approaches are often based on screening for environmental strains with protective functions. For example, Bernal et al. (59, 60) demonstrated that *P. putida* KT2440 employs its T6SS to protect *N. benthamiana* plants against *Xanthomonas campestris*. However, the introduction of biocontrol agents isolated from disparate habitats may not be able to adapt effectively within pathogen-specific niches in natural environments (10). In contrast, we present a valuable strategy that leverages the antibacterial properties of plant pathogens themselves for plant protection using *X. oryzae* as a model. This approach exploits the adaptive fitness of pathogens within their native niches, potentially yielding greater efficacy compared to introduced non-native environmental strains. This approach, alongside our previous work on avirulent *A. citrulli* for plant protection (16), exemplifies an innovative paradigm of “turning waste into treasure” by repurposing plant pathogens as biocontrol-like agents. Given the prevalence of T6SS-equipped phytopathogens (59), such a strategy offers a sustainable approach that could unlock the full potential of T6SS-mediated antibacterial functions in managing agricultural diseases. This strategy can be further strengthened by equipping these detoxified plant pathogens with designed and synthetic T6SS effectors (61). Furthermore, integrating growth-promoting gene modules into these engineered strains could empower them to serve dual roles as both biocontrol-like agents and plant growth promoters. This dual functionality provides a promising trajectory for sustainable agriculture, facilitating the simultaneous management of plant diseases while enhancing crop productivity.

## MATERIALS AND METHODS

### Strains and growth conditions

The strains, plasmids, and primers used in this study are detailed in the Appendix. *E. coli*, *V. cholerae*, and *C. rodentium* were cultured aerobically in LB medium (1% tryptone [wt/vol], 0.5% yeast extract, and 0.5% NaCl) at 37°C. *P. syringae* pv. *tomato* DC3000 and *P. syringae* pv. *tomato* PtoT1 were grown aerobically in LB medium at 28°C. *X. oryzae* was cultured in NB medium (0.3% beef extract [wt/vol], 0.1% yeast extract, 0.5% polypeptone, and 0.5% sucrose) at 28°C, and *R. solanacearum* was cultured in LBNS medium (1% tryptone [wt/vol] and 0.5% yeast extract) at 28°C. NBN medium (0.3% beef extract [wt/vol], 0.1% yeast extract, and 0.5% polypeptone) or NBS medium (0.3% beef extract [wt/vol], 0.1% yeast extract, 0.5% polypeptone, and 10% sucrose) was used for constructing gene knockout mutants of PXO99A. Antibiotic concentrations used in this study were as follows: kanamycin (50 µg/mL, except for PXO99A, 25 µg/mL) and gentamicin (20 µg/mL). Mutants were constructed by homologous double crossover using the suicide plasmid pK18mobSacB. All plasmid constructs were verified by sequencing.

### Secretion assay

Bacterial cultures were grown in NB liquid medium at 28°C with shaking at 200 rpm until an OD_600_ of 1.0 was reached. Cultures were then centrifuged at 4,500 × *g* for 4 minutes at room temperature to collect the bacterial cells. The cell pellets were resuspended in fresh NB medium and incubated at 28°C for 1 hour. The supernatant was collected by centrifugation at 10,000 × *g* for 4 minutes at room temperature, and this step was repeated twice. Cell pellets were resuspended in 2× SDS protein loading buffer to prepare whole-cell samples. Supernatant secretory proteins were enriched using the trichloroacetic acid (TCA) method as follows: TCA was added to the supernatant to a final concentration of 20% (vol/vol), and the mixture was incubated at −20°C for 30 minutes. The precipitate was collected by centrifugation at 15,000 × *g* for 30 minutes at 4°C, washed with acetone, and resuspended in 2× SDS protein loading buffer. All samples were boiled at 98°C for 10 minutes and subsequently analyzed by SDS-PAGE and Western blot.

For secretome analysis, proteins enriched through TCA precipitation were separated by SDS-PAGE, excised from the gel, and subsequently analyzed by LC-MS/MS.

### Protein abundance analysis

Mass spectrometry data and normalized protein abundances are shown in Data set S1. The data provided an initial identification of T6SS proteins. For MA plot analysis, the mean log intensity was plotted against log2(fold change) using Python to illustrate comparisons between WT (PXO99A) and Δ*tssB2* strains. All comparisons included three independent biological replicates. For volcano plot analysis, calculation of log2(fold change) and *P*-value was performed by Python using three independent biological replicates for each condition.

### Protein toxicity assay

Cells harboring different plasmids were initially cultured on LB agar plates containing appropriate antibiotics and 0.2% (wt/vol) glucose at 30°C overnight. The following day, these cells were inoculated into fresh LB liquid medium with the appropriate antibiotics and 0.2% (wt/vol) glucose and grown to an OD_600_ of 1. Serial 10-fold dilutions were then prepared and plated on LB agar plates containing the corresponding antibiotics and either 0.1% (wt/vol) L-arabinose or 0.2% (wt/vol) glucose. Each experiment was performed in at least two independent replicates, and representative results are presented.

### Protein pull-down assays

Genes were tagged with His or FLAG and cloned into the pET vector for expression. *E. coli* cultures were grown in LB medium supplemented with appropriate antibiotics until OD_600_ reached 0.6. Protein expression was induced by adding 0.1% arabinose for 3 hours at 30°C or overnight at 20°C. Following induction, cells were harvested by centrifugation and resuspended in 1 mL lysis buffer (20 mM Tris-HCl, pH 8.0, 500 mM NaCl, and 50 mM imidazole) supplemented with protease inhibitors (Thermo Fisher Scientific). Cells were lysed, and insoluble debris was removed by centrifugation. The supernatant was incubated with Ni-NTA magnetic beads (Smart Lifesciences), and bound proteins were washed four times with wash buffer (20 mM Tris-HCl, pH 8.0, 500 mM NaCl, and 50 mM imidazole) to remove non-specifically bound impurities. Target proteins were eluted using elution buffer (20 mM Tris-HCl, pH 8.0, 500 mM NaCl, and 500 mM imidazole), and the eluted samples were analyzed by Western blot.

### Bacterial competition assays

Bacterial cultures were grown in liquid culture medium at 28°C with shaking at 200 rpm until an OD_600_ of 1.0 was reached. The ratio of killer to prey cells was adjusted to 10:1, and the cells were mixed in equal volumes. The mixed cultures were then co-incubated at 28°C for specific durations: MG1655 and *X. oryzae* for 4 hours, PtoT1 and DC3000 for 2 hours, GMI1000 for 6 hours, and *V. cholerae* C6706, *E. coli* ETEC H-10407, *E. coli* DAEC 2787, and *C. rodentium* for 3 hours. Following co-culture, survival rates of killer and prey cells were determined by plating 10-fold serial dilutions on medium supplemented with the corresponding antibiotics.

### Bacterial competition assays on *N. benthamiana*

Bacterial competition experiments were conducted on 4-week-old *N. benthamiana* leaves. Killer and prey cells were adjusted to a ratio of 10:1 and mixed in equal proportions. The cell mixture was injected into the abaxial side of the leaves using a blunt syringe and marked. The inoculated plants were incubated in a plant greenhouse for 3 hours (*E. coli* ETEC H-10407 and *E. coli* DAEC 2787) and 8 hours (GMI1000). Following incubation, the infected leaves were harvested, ground in liquid nitrogen, resuspended in liquid culture medium, and serially diluted 10-fold to separate killer and prey cells for subsequent analysis.

### Bioinformatics analysis

The genome of *X. oryzae* PXO99A was obtained from the NCBI database (GenBank accession number NC_010717.2). The predicted T6SS large and small gene clusters were analyzed using HMMER and Benchling software. Statistical analysis was performed using GraphPad Prism version 9.5.1. Statistical significance was evaluated using a two-tailed Student’s *t*-test.

### Hypersensitive response assay

*X. oryzae* cultures were grown in NB medium at 28°C until reaching an OD_600_ of 1. An appropriate volume of the bacterial culture was collected by centrifugation, and the supernatant was discarded. The bacterial pellet was resuspended in a 10 mM MgCl_2_ solution to an OD_600_ of 0.8. Four-week-old *N. benthamiana* leaves were infiltrated on the abaxial side using a syringe without a needle. MgCl_2_ solution was used as a negative control. Changes in the HR on the leaves were observed daily, photographed, and documented.

### Bacterial infection assay

Mutant Δ*hrcU* was cultured in NB medium at 28°C until reaching an OD_600_ of 1, and *P. syringae* pv. *tomato* DC3000 (Pst) was cultured in LB medium at 28°C until reaching an OD_600_ of 1. Following the protocol (62) with minor modifications, large-volume inocula were prepared by diluting the bacterial cultures with 10 mM MgCl_2_ and adding 0.04% Silwet L-77. For the experiment, 4-week-old tomato (Ac) plants were used. The plants were sprayed with the following bacterial suspensions and maintained in a high-humidity environment: single cultures of Δ*hrcU* and Pst, as well as mixed cultures of Pst + Δ*hrcU*. MgCl_2_ (10 mM) was used as a negative control. Regardless of the culture type (single or mixed), the final concentration of each bacterial suspension was adjusted to an OD_600_ of 0.1. Post-inoculation, the plants were cultured in a plant greenhouse. Disease symptoms were observed and photographed daily. Disease symptoms were evaluated at 11 days post-inoculation using a 0–9 severity scale, where 0 indicates no symptoms; 1, 3, 5, and 7 indicate necrotic lesions covering approximately 25%, 50%, 75%, and 100% of the leaf area, respectively; and 9 indicates complete seedling death. The disease index (DI) was calculated using the formula: DI = ∑(*A* × *B*)/(*C* × *D*) × 100%, where A is the severity score (0, 1, 3, 5, 7, or 9), B is the number of leaves with the corresponding severity score in each treatment, C is the total number of leaves assessed in that treatment, and D is the superlative severity score.

## Data Availability

All data are available in the main text or the supplemental material.

## SUPPLEMENTAL MATERIAL

Data Set S1 aem.01021-25-s0001.xlsx Secretome data.

Supplemental material aem.01021-25-s0002.pdf Figures S1 to S3, Tables S1 to S4, and Text S1.

ASM does not own the copyrights to Supplemental Material that may be linked to, or accessed through, an article. The authors have granted ASM a non-exclusive, world-wide license to publish the Supplemental Material files. Please contact the corresponding author directly for reuse.
