# Supplementary material for: Dissecting the antibacterial functions of the T6SS-2 cluster in Xanthomonas oryzae for environment and plant protection
Source: Appl Environ Microbiol. 2025 Sep 19;91(10):e01021-25. doi: 10.1128/aem.01021-25 (PMC12542774; doi:10.1128/aem.01021-25)
Supplement: Supplemental material — Figures S1 to S3, Tables S1 to S4, and Text S1. [file aem.01021-25-s0002.pdf]

Supplementary Materials for

Dissecting the antibacterial functions of the T6SS-2 cluster in *Xanthomonas oryzae* for environment and plant protection

Zhi-Min Tan[1,2], Xin Zheng[2], Jingtong Su[2], Jin-Sheng Liu[1], Xiaoye Liang[2], Tong-Tong Pei[2], Tao Dong[2*]

*Correspondence: dongt@sustech.edu.cn

The PDF file includes:

Figures. S1 and S3
Tables. S1 to S4
Text S1

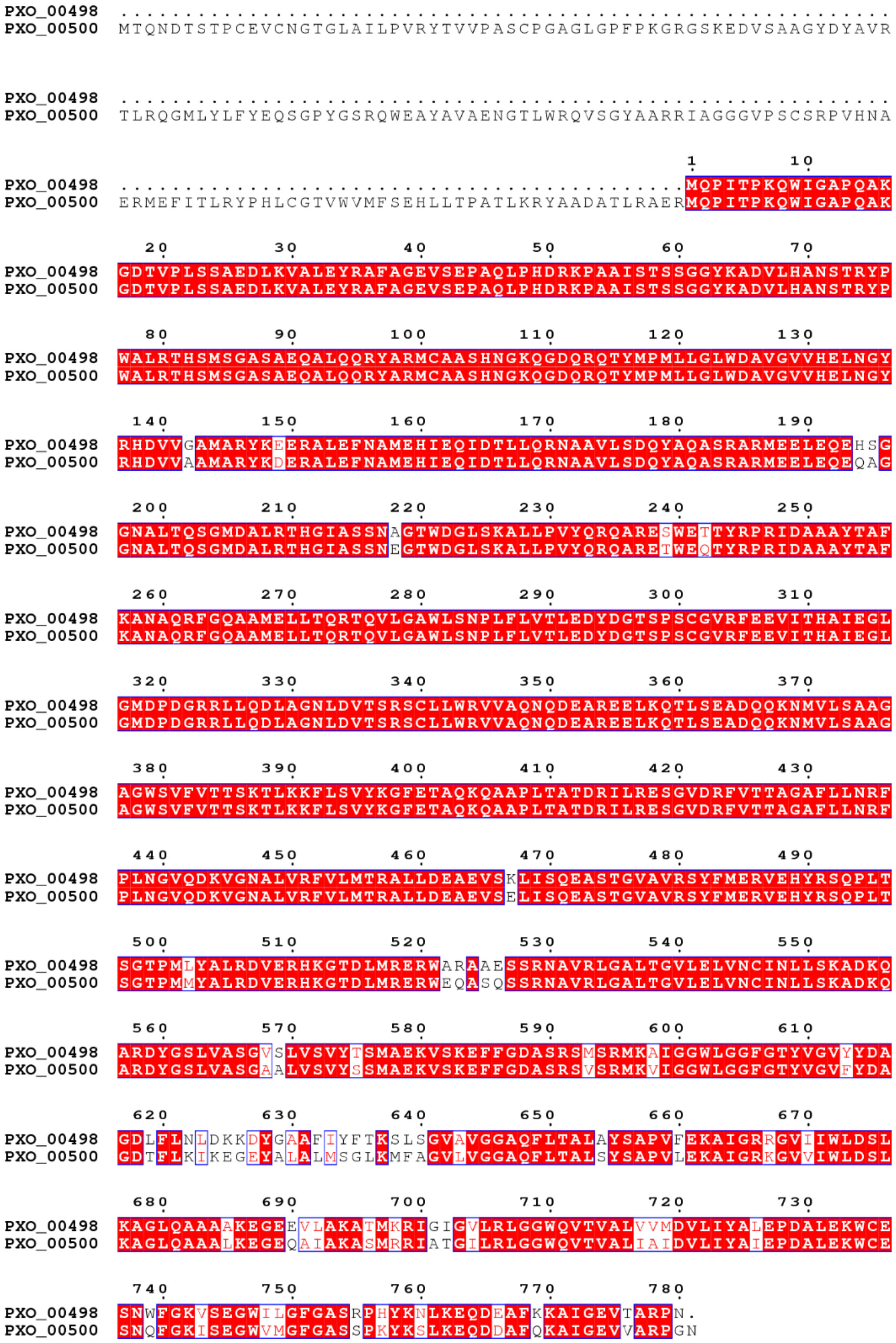

Figure S1. Sequence alignment between PXO_00498 and PXO_00500 proteins. PXO_00498 and PXO_00500 share 92% identity and 82% coverage. Protein sequences were aligned using ClustalW and visualized with ESPript (http://espript.ibcp.fr/ESPript/ESPript/). Protein sequences are provided

in Text S1.

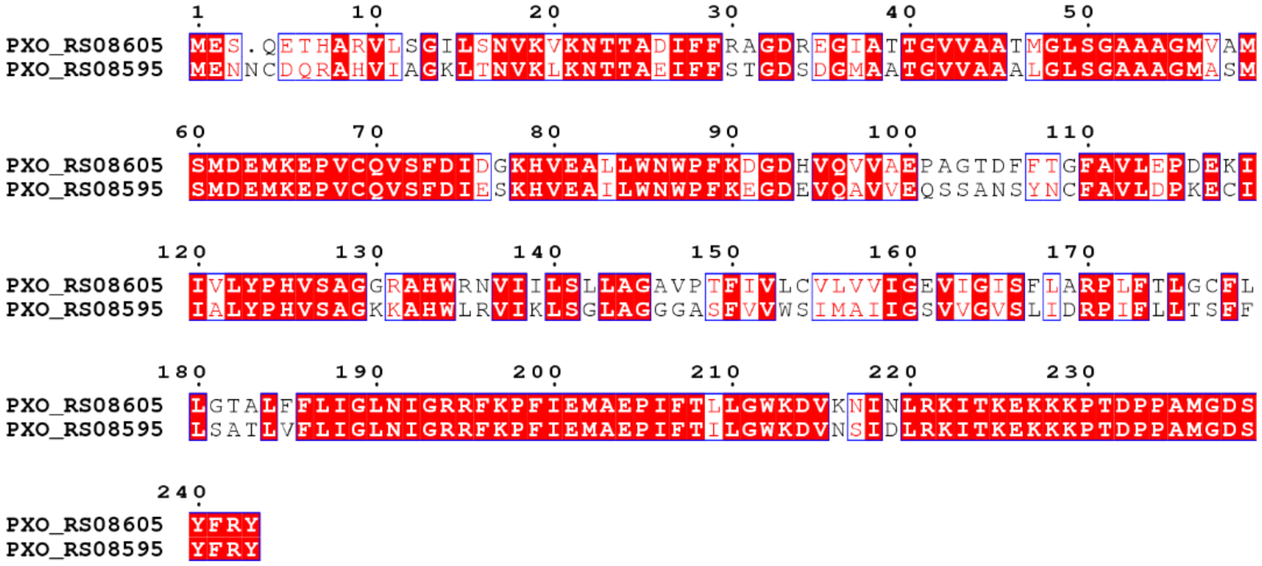

Figure S2. Sequence alignment between PXO_RS08605 and PXO_RS08595 proteins. PXO_RS08605 and PXO_RS08595 share 68% identity and 100% coverage. Protein sequences were aligned using ClustalW and visualized with ESPript (http://espript.ibcp.fr/ESPript/ESPript/). Protein sequences are provided in Text S1.

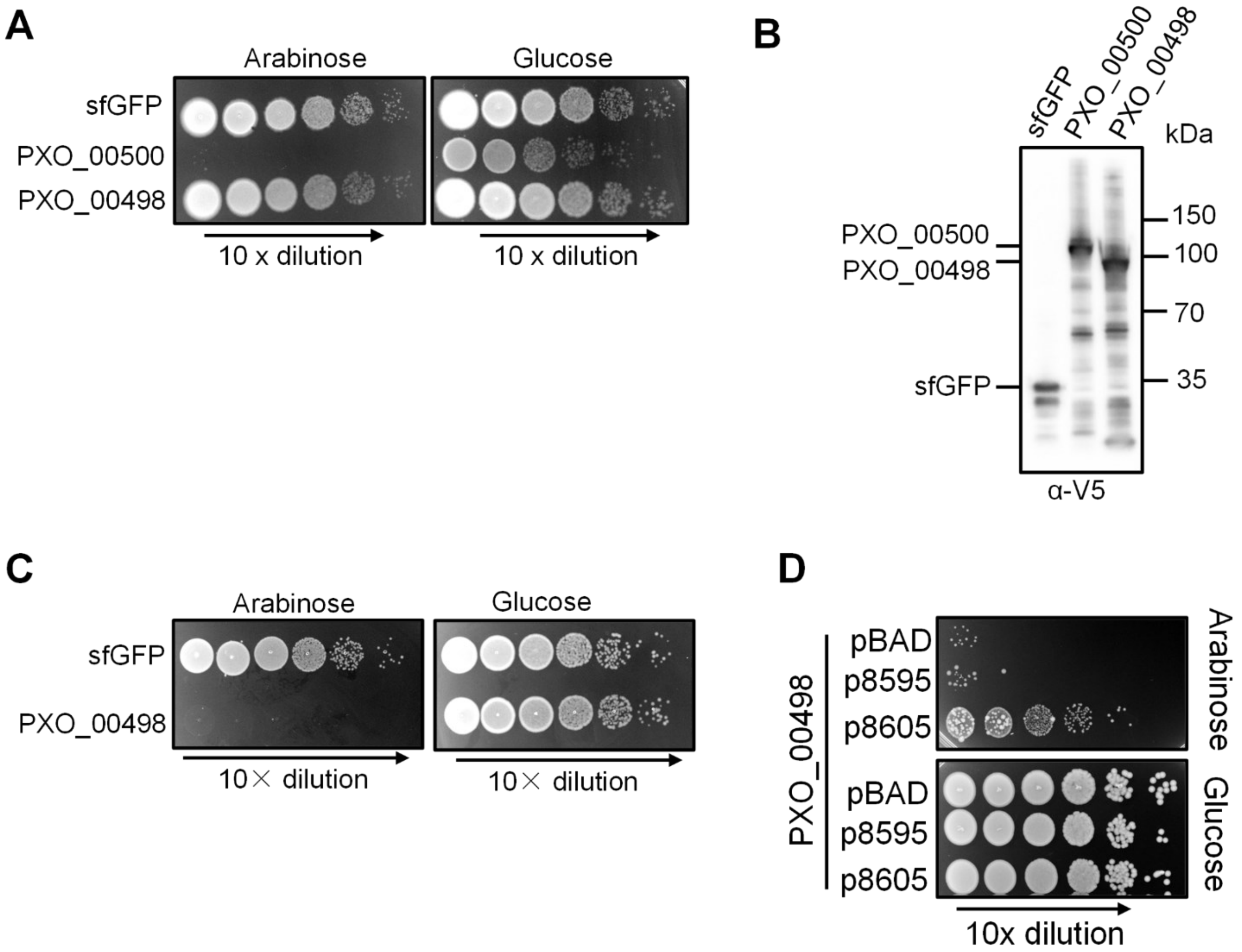

Figure S3. Cytotoxicity of effector proteins PXO_00498 and PXO_00500. (A) Toxicity of expressing PXO_00498 and PXO_00500 in *E. coli*. PXO_00498 and PXO_00500 were expressed using pBAD vectors, with an N-terminal Tat signal peptide. Expression of sfGFP, PXO_00498, and PXO_00500 was confirmed by Western blotting, as shown in (B). (C). Toxicity of expressing PXO_00498 in *Aeromonas dhakensis* SSU (SSU). PXO_00498 was expressed using pBAD vectors, with an N-terminal Sec signal peptide. (D). The toxicity of PXO_00498 with an N-terminal Sec signal peptide in SSU was evaluated using an empty vector (pBAD) or a vector carrying the immunity gene PXO_RS08595 (8595) or PXO_RS08605 (8605). All genes were cloned into pBAD, and survival of SSU cells were serially diluted and plated on medium supplemented with either the inducer arabinose or the inhibitor glucose, followed by colony enumeration.

Table S1. Size, function, and localizations of the T6SS putative effectors in PXO99A

Locus Tag	Size (AA)	Function	Effector adjacent PAAR, Hcp or VgrG
**PXO_00272**	944	Colicin N	VgrG(PXO_00270)
**PXO_02058**	671	Toxin B	VgrG(PXO_02054)
**PXO_02056**	953	Colicin IA, DUF3784	VgrG(PXO_02054)
**PXO_02034**	743	phospholipase effector	VgrG(PXO_02029)
**PXO_00500**	945	Colicin B	VgrG(PXO_00502)
**PXO_00498**	780	Colicin-E1	VgrG(PXO_00502)
**PXO_01369**	944	Colicin A	PXO_01368(DUF4123)
**PXO_RS27400**	235	Tox-REase-5	PAAR(PXO_03514)
**PXO_RS01450**	231	Tox-REase-5	PAAR(PXO_03898)
**PXO_01528**	859	VasX-N	VgrG(PXO_01531)
**PXO_03643**	714	MhpC, DUF3274	VgrG(PXO_03644)
**PXO_03639**	733	DUF3274, Abhydrolase_1	PAAR(PXO_RS25115)
**PXO_RS04170**	247	effector	VgrG(PXO_04256)
**PXO_04252**	424	Muraidase, LysM	VgrG(PXO_04253)
**PXO_05538**	1579	ABC toxin	VgrG(PXO_00004)
**PXO_02456**	185	alpha/beta hydrolase	PAAR(PXO_RS26915)
**PXO_02459**	734	DUF3274, Abhydrolase_1	PAAR(PXO_RS19665)
**PXO_02463**	715	MhpC, DUF3274	VgrG(PXO_02464)
**PXO_ 04701**	1021	toxin	VgrG(PXO_04700)
**PXO_ 02032**	299	phospholipase effector(alpha/beta hydrolase domain DUF2235)	VgrG(PXO_02029)
**PXO_ 02037**	751	Hydrolase	VgrG(PXO_02038)

Table S2. Plasmids

**Plasmid**	**Description**	**Reference**
pBBR1MCS-2	A broad-host-range cloning vector, kanamycin resistance	Lab stock
pBAD18cm	An *E. coli* protein expression vector that utilizes the araBAD promoter, chloramphenicol resistance	Lab stock
pBAD24kan-Tat signal-PXO_00500	Arabinose induces expression of PXO_00500 with a C-terminal 3V5 tag	This study
pBAD18cm-PXO_RS08595	Arabinose induces expression of PXO_RS08595 with a C-terminal FLAG tag	This study
pBAD18cm-PXO_RS08605	Arabinose induces expression of PXO_RS08605 with a C-terminal FLAG tag	This study
pBBR1MCS-5	A broad-host-range cloning vector, gentamycin resistance	Lab stock
pBAD24Kan	An *E. coli* protein expression vector that utilizes the araBAD promoter, kanamycin resistance	Lab stock

pK18mobSacB	Suicidal conjugation vector for all chromosomal allelic changes	Lab stock
pK18mobSacB-*tssB1*	Suicidal conjugation vector to construct in-frame deletion mutant of *tssB1* (PXO_00266), T6SS-1 mutant.	Lab stock
pK18mobSacB-*tssB2*	Suicidal conjugation vector to construct in-frame deletion mutant of *tssB2* (PXO_02045), T6SS-2 mutant.	Lab stock
pK18mobSacB-*hrcU*	Suicidal conjugation vector to construct in-frame deletion mutant of *hrcU* (PXO_03402), T3SS mutant.	Lab stock
pK18mobSacB-*PXO_00500-PXO_RS08605*	Suicidal conjugation vector to construct in-frame deletion mutant of *PXO_00500, PXO_RS08595, PXO_00498, PXO-RS08605*, *PXO_00500-PXO_RS08605* mutant.	This study
pBBR1MCS5-PXO_RS08595	Heterogeneously expressed PXO_RS08595	This study
pET-22b-PXO_00500-FLAG	Heterogeneously expressed PXO_00500	This study
pET28a-His-GFP	Heterogeneously expressed GFP	Lab stock
pET28a-His-PXO_00501	Heterogeneously expressed PXO_00501	This study
pET28a-His-PXO_00502	Heterogeneously expressed PXO_00502	This study
pET28a-His-PXO_RS08595	Heterogeneously expressed PXO_RS08595	This study
pET28a-His-PXO_RS08605	Heterogeneously expressed PXO_RS08605	This study
pBAD24kan-MBP-FLAG	Heterogeneously expressed MBP	Lab stock
pBAD24Kan-Sec_PXO_00498-3V5	Heterogeneously expressed PXO_00498 with a N-terminal Sec signal peptide	This study
pK18mobSacB_00502	Suicidal conjugation vector to construct in-frame deletion mutant of PXO_00502, VgrG4 mutant.	This study
pK18mobSacB_00501	Suicidal conjugation vector to construct in-frame deletion mutant of PXO_00501, chaperone mutant.	This study

Table S3. Strains

Strain	Genotype	Description	Reference
Xanthomonas oryzae pv. *oryzae* PXO99A	Parental	Parental strain	This study
	Δ*tssB1*	T6SS-1 null, in-frame deletion of *tssB1*	Lab stock
	Δ*tssB2*	T6SS-2 null, in-frame deletion of *tssB2*	Lab stock
	Δ*tssB1&B2*	T6SS-1&-2 null, in-frame deletion of *tssB1* and *tssB2*	Lab stock
	Δ*tssB2&hrcU*	in-frame deletion of *tssB2* and *hrcU*	This study
	Δ*PXO_00498 &PXO_00500*	in-frame deletion of *PXO_00500, PXO_RS08595, PXO_00498, and PXO_RS08605*	This study
	Δ*PXO_00501*	in-frame deletion of *PXO_00501*	This study
	Δ*PXO_00502*	in-frame deletion of *PXO_00502* (*vgrG4*)	This study
	Δ*hrcU*	in-frame deletion of *hrcU* (PXO_03402)	Lab stock
E. coli T-Fast		The strain used for cloning and gene expression	TIANGEN
E. coli MG1655		The strain used for a competition assay	Lab stock
E. coli WM6026		Strain used for conjugation	Lab stock
P. syringae pv. tomato DC3000		The strain used for a competition assay	Lab stock
V. cholerae C6706		The strain used for a competition assay	Lab stock
E. coli ETEC H-10407		The strain used for a competition assay	Lab stock
E. coli DAEC 2787		The strain used for a competition assay	Lab stock
C. rodentium		The strain used for a competition assay	Lab stock
GMI1000		The strain used for a competition assay	Lab stock
PtoT1		The strain used for a competition assay	Lab stock

Table S4. Primers

Primers	Sequences (5'-3')	Descriptions
PXO_Tat-00500-F	cgactgcggcgcaagcggcgatgacacagaacgacacctcaac	Forward primer to amplify PXO_00500
PXO_Tat-00500-R	ttgtggctcgacctggaaatggtaaacctattcctaatcc	Reverse primer to amplify PXO_00500
pPSV37-Tat-R	actgcggcgcaagcggcg	Reverse primer to amplify vector pBAD24Kan
pBAD24-3V5-Hifi-R	ggtaaacctattcctaatcctctccttg	Forward primer to amplify vector pBAD24Kan
PXO-RS08595-F	cgcatcggcgtctagaatggaaaataattgcgatcagcgag	Forward primer to amplify PXO_RS08595
PXO-RS08595-R	cgtcgtccttgtaatcgctgctgtagcggaagtagctgtcgcccat	Reverse primer to amplify PXO_RS08595
pBAD24-Sec-Hifi-r1	tttccattctagacgccgatgcgctaaacgc	Reverse primer to amplify vector pBAD24cm
pBAD24cm-Sec-FLAG-F	agcagcgattacaaggacgacg	Forward primer to amplify vector pBAD24cm
PXO-RS08605-F	cgcatcggcgtctagaatggaaagccaggaaacgcatg	Forward primer to amplify PXO_RS08605
PXO-RS08605-R	cgtcgtccttgtaatcgctgctgtagcggaagtagctgtcgcccat	Reverse primer to amplify PXO_RS08605
pK18mobSacB-R-Hifi	ccgagctcgaattcgtaatc	Reverse primer to amplify vector pK18mobSacB for constructing deleting plasmid
pK18mobSacB-F-Hifi	tcgttttacaacgtcgtgact	Forward primer to amplify vector pK18mobSacB for constructing deleting plasmid
tssB1-KO5	agcctcgcagctctgcaact	Forward primer for knockout validation
tssB1-KO1	gattacgaattcgagctcgggcactgcacgtggtgtacca	Forward primer to amplify upstream of *tssB1* for constructing deleting plasmid
tssB1-KO2	cttcgacttgggagcctcgacgtcgtactcgatctg	Reverse primer to amplify upstream of *tssB1* for constructing deleting plasmid
tssB1-KO3	gagtacgacgtcgaggctcccaagtcgaaggatgc	Forward primer to amplify downstream of *tssB1* for constructing deleting plasmid
tssB1-KO4	agtcacgacgttgtaaaacgacctcggtcgggcacttcat	Reverse primer to amplify downstream of *tssB1* for constructing deleting plasmid
tssB1-KO6	cgatgtaccagcggcatcag	Reverse primer for knockout validation
hrcU-KO1	gattacgaattcgagctcggcaacttgcgctgatcgttg	Forward primer to amplify upstream of *hrcU* for constructing deleting plasmid
hrcU-KO2	ggaagaaaaagccctgccatgctaggag	Reverse primer to amplify upstream of *hrcU* for constructing deleting plasmid

hrcU-KO3	atggcagggctttttcttccgacattgccttat	Forward primer to amplify downstream of *hrcU* for constructing deleting plasmid
hrcU-KO4	agtcacgacgttgtaaaacgaagctcgtgcatcaacttgatct	Reverse primer to amplify downstream of *hrcU* for constructing deleting plasmid
hrcU-KO5	gcgaagtcaggagcggtttt	Forward primer for knockout validation
hrcU-KO6	cgtctgcacggcgttctt	Reverse primer for knockout validation
tssB2- KO5	agcggataacaatttcacacagga	Forward primer for knockout validation
tssB2- KO1	cggtacccggggatcctctagaaggtcgctgagttccttctc	Forward primer to amplify upstream of *tssB2* for constructing deleting plasmid
tssB2- KO2	atggctaagaaggaagaaaaggagaagtaagccatgg	Reverse primer to amplify upstream of *tssB2* for constructing deleting plasmid
tssB2- KO3	ttacttctccttttcttccttcttagccattggtg	Forward primer to amplify downstream of *tssB2* for constructing deleting plasmid
tssB2- KO4	tgcctgcaggtcgactctagaggcggctacaataagaacca	Reverse primer to amplify downstream of *tssB2* for constructing deleting plasmid
tssB2- KO6	cgccagggttttcccagtcacgac	Reverse primer for knockout validation
pBBR-08595-F	gctctagaactagtggatccttagtagcggaagtagctgtcgccc	Forward primer to amplify PXO_RS08605
pBBR-08595-R	cgacggtatcgataagcttgatggaaaataattgcgatcagcga	Reverse primer to amplify PXO_RS08605
PXO_00500-RS08605-KO5	gcgatccgacattggtcaa	Forward primer for knockout validation
PXO_00500-RS08605-KO1	gattacgaattcgagctcggaagatccacaagctgccgg	Forward primer to amplify upstream of PXO_00500-RS08605 for constructing deleting plasmid
PXO_00500-RS08605-KO2	gaagtagctgtcgccggtgccgttgcagacctc	Reverse primer to amplify upstream of PXO_00500-RS08605 for constructing deleting plasmid
PXO_00500-RS08605-KO3	gtctgcaacggcaccggcgacagctacttccgctacta	Forward primer to amplify downstream of PXO_00500-RS08605 for constructing deleting plasmid
PXO_00500-RS08605-KO4	agtcacgacgttgtaaaacgaccaccagaccttcgtgttgc	Reverse primer to amplify downstream of PXO_00500-RS08605 for constructing deleting plasmid
PXO_00500-RS08605-KO6	ggccactcgatcagcagc	Reverse primer for knockout validation
PXO_00500-F	tttaagaaggagatatacatatgacacagaacgacacctcaacg	Forward primer to amplify PXO_00500
PXO_00500-R	tcatcgtcgtccttgtaatcatttccaggtcgagccacaa	Reverse primer to amplify PXO_00500
pET22b-FLAG-Hifi-F	gattacaaggacgacgatgacaag	Forward primer to amplify vector pET22b

pET22b-FLAG-Hifi-R	atgtatatctccttcttaaagttaaacaaaat	Reverse primer to amplify vector pET22b
PXO_00501-F	gtgccgcgcggcagccatatgatggacgtgaacgaacgcctctac	Forward primer to amplify PXO_00501
PXO_00501-R	ctcgagtgcggccgcaagctttcatggcgtcatgcgctcc	Reverse primer to amplify PXO_00501
PXO_00502-F	gtgccgcgcggcagccatatgatggacccggtcgccacagtact	Forward primer to amplify PXO_00502
PXO_00502-R	ctcgagtgcggccgcaagctttcaccctaggtctgcggcggcttc	Reverse primer to amplify PXO_00502
PXO_RS08595-F	tgccgcgcggcagccatatgatggaaaataattgcgatcagcg	Forward primer to amplify PXO_RS08595
PXO_RS08595-R	tcgagtgcggccgcaagcttttagtagcggaagtagctgtcgc	Reverse primer to amplify PXO_RS08595
PXO_RS08605-F	tgccgcgcggcagccatatgatggaaagccaggaaacgcat	Forward primer to amplify PXO_RS08605
PXO_RS08605-R	tcgagtgcggccgcaagcttttagtagcggaagtagctgtcgcc	Reverse primer to amplify PXO_RS08605
PXO_Tat-00498-F	agcggcgatgcaaccgatcacgccc	Forward primer to amplify PXO_00498
PXO_Tat-00498-R	gtcacggctcgccccaacggtaaacct	Reverse primer to amplify PXO_00498
pBAD24-3V5-Hifi-F1	ctcgccccaacggtaaacctattcctaatcctctccttg	Forward primer to amplify vector pBAD24
pBAD24-3V5-Hifi-R1	actgcggcgcaagcggcgatgcaaccgatca	Reverse primer to amplify vector pBAD24
PXO_00502-KO5	aacacccggcacatcgcg	Forward primer for knockout validation
PXO_00502-KO1	gattacgaattcgagctcgggcggtgggcttgcatgagg	Forward primer to amplify upstream of PXO_00502 for constructing deleting plasmid
PXO_00502-KO2	ctaggtctgcggcgaccgggtccataggtg	Reverse primer to amplify upstream of PXO_00502 for constructing deleting plasmid
PXO_00502-KO3	cccggtcgccgcagacctagggtgagacctctcatgg	Forward primer to amplify downstream of PXO_00502 for constructing deleting plasmid
PXO_00502-KO4	agtcacgacgttgtaaaacgaggccaggcaggcgatatcg	Reverse primer to amplify downstream of PXO_00502 for constructing deleting plasmid
PXO_00502-KO6	cgcacgacctgcagcgtc	Reverse primer for knockout validation
PXO_00501-KO1	gattacgaattcgagctcggtgtccacgcaggcctacgg	Forward primer to amplify upstream of PXO_00501 for constructing deleting plasmid
PXO_00501-KO2	tcatgcgctcgcgttcgttcacgtccatgagag	Reverse primer to amplify upstream of PXO_00501 for constructing deleting plasmid
PXO_00501-KO3	gaacgaacgcgagcgcatgacgccatgacac	Forward primer to amplify downstream of PXO_00501 for constructing deleting plasmid

PXO_00501-KO4	agtcacgacgttgtaaaacgagcagcatcggcatgtaggtctgt	Reverse primer to amplify downstream of PXO_00501 for constructing deleting plasmid
PXO_00501-KO5	caacctgcgcgaaggcg	Forward primer for knockout validation
PXO_00501-KO6	ccgttgagctcgtgcaccac	Reverse primer for knockout validation

Text S1. Amino acid sequencce of PXO_00500, PXO_00498, PXO_RS08596, and PXO_ RS08605

>PXO_00498
MQPITPKQWIGAPQAKGDTVPLSSAEDLKVALEYRAFAGEVSEPAQLPHDRKPAAISTSSGG
YKADVLHANSTRYPWALRTHSMSGASAEQALQQRYARMCAASHNGKQGDQRQTYMPML
LGLWDAVGVVHELNGYRHDVVGAMARYKEERALEFNAMEHIEQIDTLLQRNAAVLSDQY
AQASRARMEELEQEHSGGNALTQSGMDALRTHGIASSNAGTWDGLSKALLPVYQRQARE
SWETTYRPRIDAAAYTAFKANAQRFGQAAMELLTQRTQVLGAWLSNPLFLVTLEDYDGTS
PSCGVRFEEVITHAIEGLGMDPDGRRLLQDLAGNLDVTSRSCLLWRVVAQNQDEAREELK
QTLSEADQQKNMVLSAAGAGWSVFVTTSKTLKKFLSVYKGFETAQKQAAPLTATDRILRES
GVDRFVTTAGAFLLNRFPLNGVQDKVGNALVRFVLMTRALLDEAEVSKLISQEASTGVAVR
SYFMERVEHYRSQPLTSGTPMLYALRDVERHKGTDLMRERWARAAESSRNAVRLGALTGV
LELVNCINLLSKADKQARDYGSLVASGVSLVSVYTSMAEKVSKEFFGDASRSMSRMKAIGG
WLGGFGTYVGVYYDAGDLFLNLDKKDYGAAFIYFTKSLSGVAVGGAQFLTALAYSAPVFE
KAIGRRGVIIWLDSLKAGLQAAAAKEGEEVLAKATMKRIGIGVLRLGGWQVTVALVVMDV
LIYALEPDALEKWCESNWFGKVSEGWILGFGASRPHYKNLKEQDEAFKKAIGEVTARPN
>PXO_00500
MTQNDTSTPCEVCNGTGLAILPVRYTVVPASCPGAGLGPFPKGRGSKEDVSAAGYDYAVRT
LRQGMLYLFYEQSGPYGSRQWEAYAVAENGTLWRQVSGYAARRIAGGGVPSCSRPVHNAE
RMEFITLRYPHLCGTVWVMFSEHLLTPATLKRYAADATLRAERMQPITPKQWIGAPQAKGD
TVPLSSAEDLKVALEYRAFAGEVSEPAQLPHDRKPAAISTSSGGYKADVLHANSTRYPWAL
RTHSMSGASAEQALQQRYARMCAASHNGKQGDQRQTYMPMLLGLWDAVGVVHELNGY
RHDVVAAMARYKDERALEFNAMEHIEQIDTLLQRNAAVLSDQYAQASRARMEELEQEQA
GGNALTQSGMDALRTHGIASSNEGTWDGLSKALLPVYQRQARETWEQTYRPRIDAAAYTA
FKANAQRFGQAAMELLTQRTQVLGAWLSNPLFLVTLEDYDGTSPSCGVRFEEVITHAIEGL
GMDPDGRRLLQDLAGNLDVTSRSCLLWRVVAQNQDEAREELKQTLSEADQQKNMVLSAA
GAGWSVFVTTSKTLKKFLSVYKGFETAQKQAAPLTATDRILRESGVDRFVTTAGAFLLNRF
PLNGVQDKVGNALVRFVLMTRALLDEAEVSELISQEASTGVAVRSYFMERVEHYRSQPLTS
GTPMMYALRDVERHKGTDLMRERWEQASQSSRNAVRLGALTGVLELVNCINLLSKADKQ
ARDYGSLVASGAALVSVYSSMAEKVSKEFFGDASRSVSRMKVIGGWLGGFGTYVGVFYD
AGDTFLKIKEGEYALALMSGLKMFAGVLVGGAQFLTALSYSAPVLEKAIGRKGVVIWLDSL
KAGLQAAALKEGEQAIAKASMRRIATGILRLGGWQVTVALIAIDVLIYAIEPDALEKWCESN
QFGKISEGWVMGFGASSPKYKSLKEQDDAFQKAIGEVVARPGN
>PXO_RS08605
MESQETHARVLSGILSNVKVKNTTADIFFRAGDREGIATTGVVAATMGLSGAAAGMVAMS
MDEMKEPVCQVSFDIDGKHVEALLWNWPFKDGDHVQVVAEPAGTDFFTGFAVLEPDEKII
VLYPHVSAGGRAHWRNVIILSLLAGAVPTFIVLCVLVVIGEVIGISFLARPLFTLGCFLLGTAL
FFLIGLNIGRRFKPFIEMAEPIFTLLGWKDVKNINLRKITKEKKKPTDPPAMGDSYFRY
>PXO_RS08595
MENNCDQRAHVIAGKLTNVKLKNTTAEIFFSTGDSDGMAATGVVAAALGLSGAAAGMAS
MSMDEMKEPVCQVSFDIESKHVEAILWNWPFKEGDEVQAVVEQSSANSYNCFAVLDPKECI
IALYPHVSAGKKAHWLRVIKLSGLAGGGASFVVWSIMAIIGSVVGVSLIDRPIFLLTSFFLSA
TLVFLIGLNIGRRFKPFIEMAEPIFTILGWKDVNSIDLRKITKEKKKPTDPPAMGDSYFRY
